# Improved therapy for medulloblastoma: targeting hedgehog and PI3K-mTOR signaling pathways in combination with chemotherapy

**DOI:** 10.18632/oncotarget.24618

**Published:** 2018-03-30

**Authors:** Nagendra K. Chaturvedi, Matthew J. Kling, Don W. Coulter, Timothy R. McGuire, Sutapa Ray, Varun Kesherwani, Shantaram S. Joshi, J. Graham Sharp

**Affiliations:** ^1^ Departments of Pediatrics, Hematology and Oncology, University of Nebraska Medical Center, Omaha, NE 69198, USA; ^2^ Genetics, Cell Biology and Anatomy, University of Nebraska Medical Center, Omaha, NE 69198, USA; ^3^ Pharmacy Practice, University of Nebraska Medical Center, Omaha, NE 69198, USA; ^4^ Pathology and Microbiology, University of Nebraska Medical Center, Omaha, NE 69198, USA

**Keywords:** medulloblastoma, hedgehog/PI3K-mTOR pathway, MYC, small molecule inhibitors, chemotherapy

## Abstract

Aberrant activation and interactions of hedgehog (HH) and PI3K/AKT/mTOR signaling pathways are frequently associated with high-risk medulloblastoma (MB). Thus, combined targeting of the HH and PI3K/AKT/mTOR pathways could be a viable therapeutic strategy to treat high-risk patients. Therefore, we investigated the anti-MB efficacies of combined HH inhibitor Vismodegib and PI3K-mTOR dual-inhibitor BEZ235 together or combined individually with cisplatin against high-risk MB. Using non-MYC- and MYC-amplified cell lines, and a xenograft mouse model, the *in vitro* and *in vivo* efficacies of these therapies on cell growth/survival and associated molecular mechanism(s) were investigated. Results showed that combined treatment of Vismodegib and BEZ235 together, or with cisplatin, significantly decreased MB cell growth/survival in a dose-dependent-fashion. Corresponding changes in the expression of targeted molecules following therapy were observed. Results demonstrated that inhibitors not only suppressed MB cell growth/survival when combined, but also significantly enhanced cisplatin-mediated cytotoxicity. Of these combinations, BEZ235 exhibited a significantly greater efficacy in enhancing cisplatin-mediated MB cytotoxicity. Results also demonstrated that the MYC-amplified MB lines showed a higher sensitivity to combined therapies compared to non-MYC-amplified cell lines. Therefore, we tested the efficacy of combined approaches against MYC-amplified MB growing in NSG mice. *In vivo* results showed that combination of Vismodegib and BEZ235 or their combination with cisplatin, significantly delayed MB tumor growth and increased survival of xenografted mice by targeting HH and mTOR pathways. Thus, our studies lay a foundation for translating these combined therapeutic strategies to the clinical setting to determine their efficacies in high-risk MB patients.

## INTRODUCTION

Medulloblastoma (MB) is the most prevalent pediatric brain tumor and one of the leading causes of brain cancer deaths in children [1]. Recent genomic studies in primary MB have led to a classification of the disease into four molecular subtypes: WNT, sonic hedgehog (SHH), Group 3 (MYC amplification) and Group 4 (heterogeneous genes). The WNT-MBs subgroup displays the most favorable patient outcomes, while Group 3 and Group 4 MB patients demonstrate the poorer survival outcomes [1–3]. Of these four types, SHH-MBs are most common in infants and adults [4, 5]. Small molecule inhibitors have been developed that largely target the SMO component of SHH signaling [4–6]. However, current treatment with these inhibitors has demonstrated limited efficacy due to drug resistance [6–8]. Understanding the diverse events driving tumor progression and recurrence is necessary for identifying novel, targeted therapeutics and improving survival of patients with MB [9, 10].

The HH/GLI signaling pathway has multiple roles in the initiation and progression of many human cancers, in that it regulates oncogenic events such as proliferation, survival, metastasis, and cancer stem cell function [4, 11]. The activation and regulation of HH signaling is a complex process that occurs at multiple levels within a signal cascade. Canonical HH signaling is activated on the binding of the SHH ligand to its receptor patched (PTCH). HH interaction with PTCH attenuates the inhibitory effect on the transmembrane protein smoothened (SMO). De-repressed SMO triggers the GLI transcription factors and once activated, GLIs turn on transcriptional activity that regulates those genes involved in processes such as cell cycle, survival, metabolism, and stemness [12, 13]. In addition to this canonical regulation of HH signaling, the noncanonical (SMO-independent) regulation of this signaling often involves crosstalk and interaction with other oncogenic signaling pathways such as PI3K/AKT/mTOR and RAS/MAPK, which also modulates the output of HH signaling [14–18]. Several studies have shown that HH signaling regulates protein translation components via PI3K-mTOR signaling [13, 18]. In addition, translational regulators have been shown to modulate HH function [19–21]. The overexpression and activation of PI3K-mTOR signaling, including translation pathway components, frequently occurs in HH-driven MB tumors resistant to SMO inhibitors; the crosstalk and interaction between HH and PI3K-mTOR might be a cause of this tumor resistance [22–24]. Emerging evidence based on several preclinical mouse model studies also demonstrated the activation and importance of PI3K-mTOR signaling in a MYC-driven (Group 3) MB development [25–31], suggesting that PI3K-mTOR signaling not only plays a role in SHH-driven MB tumor progression, but also is equally important for MYC-driven MB development. In addition, the MYC-driven MBs have been shown to be resistant to HH pathway inhibitors due to activation of PI3K-mTOR signaling in preclinical mouse models. Therefore, combined targeting of HH/PI3K-mTOR signaling pathways is viable and logical therapeutic strategy for the treatment of MB patients with HH/MYC-driven. To that end, a number of small molecule inhibitors targeting these key pathways have been developed [5, 18].

The small molecule inhibitors Vismodegib and BEZ235 are orally bioavailable compounds currently in phase I/II clinical trials for several advanced solid tumors. Vismodegib, a selective HH (SMO) pathway inhibitor, was approved by the U.S. Food and Drug Administration (FDA) for treatment of advanced basal cell carcinoma [32]. BEZ235 is an ATP-competitive and potent dual PI3K and mTOR1/2 pathway inhibitor [33]. Both inhibitors can cross the blood-brain barrier and have excellent pharmacokinetic profiles and antitumor activities with a good safety profile in patients with various advanced solid tumors, making them potential candidates for MB therapeutics [32–36].

In the present study, we investigated the combined efficacy of Vismodegib and BEZ235 against HH/MYC-driven MB. We observed that combination of Vismodegib and BEZ235 significantly inhibits MB cell growth and survival by targeting associated pathways. We also demonstrated that inhibitors not only inhibit MB cell growth/survival when combined, but also significantly enhanced cisplatin-mediated cytotoxicity. Our i*n vivo* results using NSG xenografts showed that combination of Vismodegib and BEZ235 or their combination individually with cisplatin significantly decreased MB tumor growth and increased survival of xenograft mice by targeting HH and mTOR pathways. The combined results of cell-based and *in vivo* studies suggest that Vismodegib combined with BEZ235 exhibited sufficient anti-tumor activity against HH/MYC-driven MB at clinically achievable *in vivo* concentrations.

## RESULTS

### Single agent inhibitory efficacy of Vismodegib, BEZ235 and cisplatin on MB cell growth

**Figure 1:**
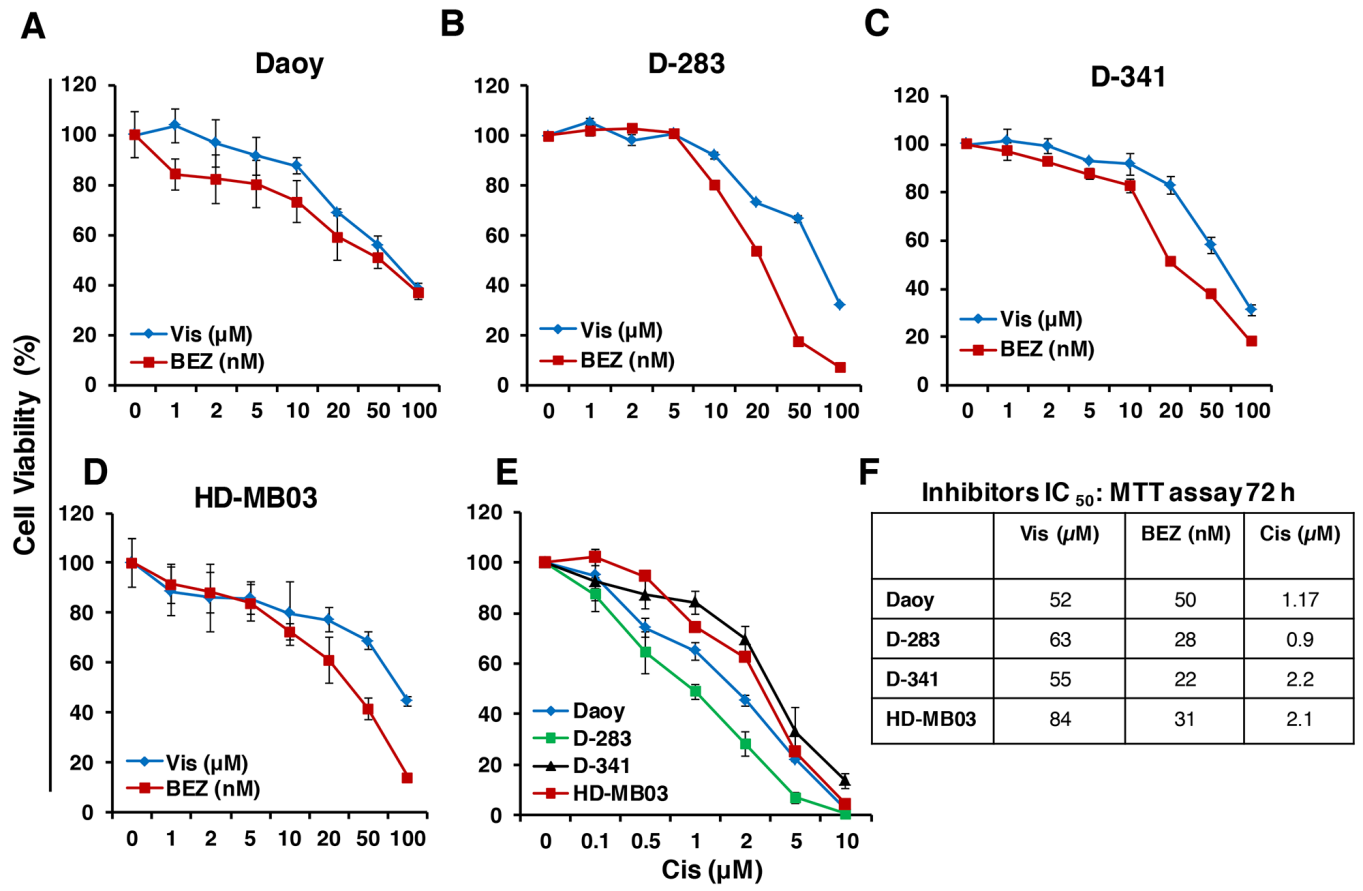
Single agent effects of Vismodegib (Vis), BEZ235 (BEZ) and cisplatin (Cis) on MB cell growth *in vitro* Exponentially growing cells of each MB line were treated with increasing concentrations of Vis (1-100 μM), BEZ (1-100 nM) and Cis (0.1-10 μM) or DMSO (vehicle, 0.05%)) in 96-well plates and the growth of these cells was determined at 72 hours using MTT assay. The percentage of cell viability is relative to control vehicle-treated cells. The values represent the means ± SD from four wells of 96-well plates. **(A-D)** Show the dose-response effects of inhibitors Vis and BEZ and **(E)** shows the dose-response effect of Cis in all four MB lines as indicated. **(F)** IC_50_ values of the indicated inhibitors and Cis in four MB cell lines. Note: *The agents Vismodegib, BEZ235 and cisplatin are abbreviated as Vis, BEZ and Cis, respectively, throughout all Figures.*

To determine the single agent growth inhibitory effect of HH pathway inhibitor Vismodegib, PI3K-mTOR pathway dual inhibitor BEZ235 and chemotherapy cisplatin against HH/MYC-driven MB *in vitro*, the HH-derived MB cell line Daoy and three MYC-amplified MCL cell lines D-283, D-341 and HD-MB03 [37, 38] were incubated individually with Vismodegib (1-100 μM), BEZ235 (1-100 nM) and cisplatin (0.1-10 μM) in a dose-dependent manner for 72 hours, and the growth of the cells was assessed using MTT assays. The concentrations used for those inhibitors were chosen from our observations and published studies [32, 33, 36]. The MTT result shown in Figure 1 clearly showed a dose-dependent growth inhibition of all MB lines following treatment with all three inhibitors including cisplatin. The IC_50_ of each inhibitorwas within clinically achievable range. BEZ235, as single agent, showed superior efficacy inhibiting MB cell growth at nM potency compared to Vismodegib μM activity. Vismodegib was found to be less effective with varied IC_50_ (52 to 84 μM) efficacies among MB lines. However, BEZ235 was able to inhibit growth of MYC-amplified MB cells with lower IC_50_ (22 to 31 nM) and showed efficacy against non-MYC amplified (Daoy) cells with approximately 2-fold higher IC_50_ (50 nM), suggesting higher sensitivity of MYC-amplified MB cells to BEZ235. We also determined the IC_50_ of cisplatin on MB cell growth. Our results with cisplatin cytotoxicity in MB cells showed increased (~2 fold) IC_50_ in highly MYC-amplified (D-341, HD-MB03) MB cells, compared to moderate MYC-amplified D-283 and non-MYC-amplified Daoy (HH-driven) MB cell lines (Figure 1F). The increased IC_50_ values of cisplatin in highly MYC-amplified cells indicated relatively chemoresistance nature of these cells. Overall, these results demonstrated superior efficacy of the PI3K-mTOR dual inhibitor BEZ235 and least efficacy of cisplatin against MYC-amplified MB cells *in vitro*.

As Vismodegib was able to show limited anti-MB efficacy with high IC_50_ (>50 μM), in order to investigate the specificity of Vismodegib on SHH signaling, we determined the IC_50s_ and kinetics of Vismodegib on GLI1 expression and correlated with cell growth inhibition in MB cells. Our results with GLI1 qPCR experiment in SHH-driven Daoy cells demonstrated that although Vismodegib showed relatively lower IC_50s_ on GLI1 expression compared to cell growth inhibition, the kinetics of GLI1 inhibition by Vismodegib followed the similar pattern as in cell growth inhibition (Supplementary Figure 1A). Interestingly, we observed that MYC-driven MB cell line HD-MB03 was less responsive to Vismodegib as it exhibited greater IC_50_ (>80 μM) in inhibiting GLI1 expression and consistent with cell growth inhibition in these experiments compared to SHH-driven Daoy cells. These results were also consistent with our observation of the significantly higher (10-12 fold) expression of GLI1 in Daoy cells compared to MYC-driven HD-MB03 cells (Supplementary Figure 1B), indicating overexpressed SHH components making Daoy cells more responsive to SHH inhibitor. Together, these results suggest that Vismodegib specifically targets SHH signaling with high doses and therefore, inhibits MB cell growth.

### Combination efficacy of Vismodegib and BEZ235 on MB cell growth and apoptosis

**Figure 2:**
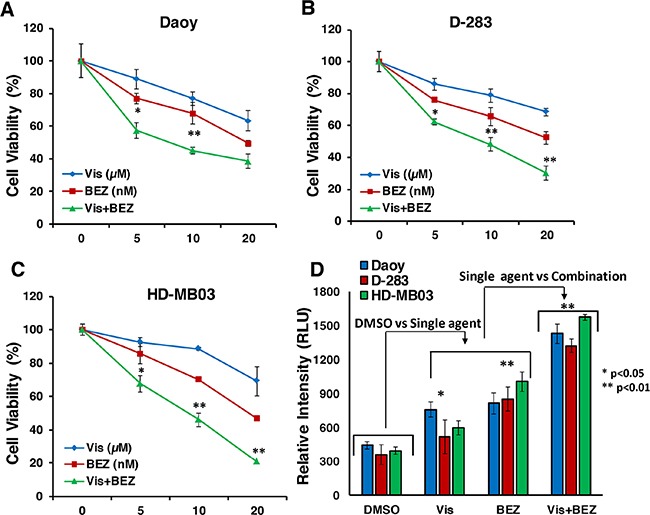
Combination effect of Vis and BEZ on MB cell growth/survival **(A-C)** MTT assay showing the single agents and combination effects of Vis and BEZ on MB cell line growth (as indicated) at 72 hours in a dose-dependent manner. The values represent the means ± SD from four wells of 96-well plates. **(D)** Caspase 3/7 activity assay based on luminescence, shows apoptotic induction of MB cells treated with Vis (20 μM) and BEZ (20 nM) alone or combined for 72 hours. The values represent the means ± SD. ^*^p<0.05; ^**^p<0.01.

To examine the combined efficacy of Vismodegib and BEZ235 to inhibit growth and induce apoptosis of SHH/MYC-driven MB cells *in vitro*, MB cells (Daoy, HD-MB03) were treated with inhibitors alone or were combined in a dose-dependent fashion for 72 h. Growth inhibition and apoptotic induction of MB cells were assessed using MTT and caspase 3/7 activity assays, respectively. MTT result showed dose-dependent growth inhibition of MB cells following treatment with both inhibitors as shown in Figure 2A-2C. However, BEZ235, as a single agent at nM potency, showed greater efficacy in inhibiting the growth of both HH-derived and MYC-amplified MB lines, compared to Vismodegib activity, suggesting the role of the PI3K-mTOR pathway in SHH/MYC-driven MB progression. The combination of Vismodegib and BEZ235 significantly enhanced growth inhibition of both MB cell lines in a dose-dependent manner. In addition, our combination index (CI) analyses using Chou-Talalay method confirmed that this combined inhibition of MB cell growth was due to synergistic (CI <0.8) interactions between Vismodegib and BEZ235 (Table 1). Our results with apoptosis analyses (Figure 2D) using caspase 3/7 assay demonstrated a significantly enhanced induction of apoptosis with the combined inhibitors in both MB lines, and showed consistency with MTT growth study. These results suggest that the combination of these two inhibitors suppresses growth and/or survival of HH/MYC-driven MB cells *in vitro*.

**Table 1:** Combination index (CI) analyses of the inhibitors in MB cell lines

Vismodegib (Vis) vs BEZ235 (BEZ)								
Daoy			D-283			HD-MB03		
Vis (μM)	BEZ (nM)	CI	Vis (μM)	BEZ (nM)	CI	Vis (μM)	BEZ (nM)	CI
5	5	0.768	5	5	0.795	5	5	0.714
10	10	0.685	10	10	0.758	10	10	0.658
20	20	0.827	20	20	0.708	20	20	0.608
**Vismodegib (Vis) vs cisplatin (Cis)**								
**Daoy**			**D-283**			**HD-MB03**		
**Vis (μM)**	**Cis (μM)**	**CI**	**Vis (μM)**	**Cis (μM)**	**CI**	**Vis (μM)**	**Cis (μM)**	**CI**
5	0.5	1.122	5	0.5	1.134	5	0.5	0.878
10	1	0.818	10	1	1	10	1	0.958
20	1.5	0.908	20	1.5	0.813	20	1.5	0.883
**BEZ235 (BEZ) vs cisplatin (Cis)**								
**Daoy**			**D-283**			**HD-MB03**		
**BEZ (nM)**	**Cis (μM)**	**CI**	**BEZ (nM)**	**Cis (μM)**	**CI**	**BEZ (nM)**	**Cis (μM)**	**CI**
5	0.5	0.78	5	0.5	0.886	5	0.5	0.347
10	1	0.545	10	1	0.814	10	1	0.321
20	1.5	0.523	20	1.5	0.655	20	1.5	0.278

MB cells were treated for 72 hours with indicated concentrations of inhibitors. Cell growth was determined using MTT assay. CI was calculated by CalcuSyn software as described in Materials and methods for data shown in Figures 2 and 4. CI < 0.9 indicates synergism, 0.9–1.1 additivity and >1.1 antagonism.

To address the toxicity issue of the inhibitors, we also determined their effects on the viability of normal peripheral blood mononuclear cells (PBMCs) of healthy donors. The results of MTT assay clearly showed no significant effects of Vismodegib (1-200 μM ranges) and BEZ235 (1-200 nM ranges) alone or combined (as in this study) on the viability of normal PBMCs (Supplementary Figure 2), suggesting inhibitors specificity to kill tumor cells only, not normal or untransformed cells.

### Combination effects of Vismodegib and BEZ235 on associated pathways/molecules

**Figure 3:**
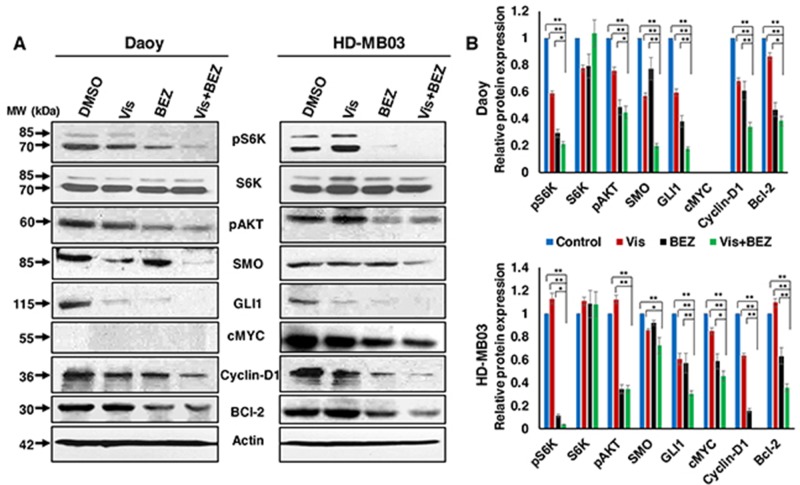
Combination efficacy of Vis and BEZ on associated pathways/molecules **(A)** Daoy and HD-MB03 MB cells were treated with 20 μM Vis and 20 nM BEZ alone or combined for 24 hours and the expression of PI3K-mTOR and HH pathways associated key molecules were determined using western blot analyses. β-Actin was used as a loading control in these experiments. **(B)** Bar graphs show the quantification of protein expression relative to the control (DMSO) in Daoy and HD-MB03 MB cells after β-Actin normalization using the Image-J software. ^*^p<0.05; ^**^p<0.01.

We next asked whether the molecular mechanism(s) causing inhibitor efficacy abrogates the target pathways/molecules. We determined the expression and/or activation of inhibitor-specific pathways/molecules by western blotting. Our results shown in Figure 3 demonstrated that BEZ235 combined with Vismodegib significantly downregulated the phosphorylated levels of PI3K-mTOR pathway molecules (S6K, AKT) and the expression of HH pathway components (SMO, GLI1) in both SHH- and MYC-driven MB cells. Subsequently, the combination significantly decreased the expression levels of downstream target molecules such as c-MYC, Cyclin D1, and Bcl-2. We could not detect expression of c-MYC protein in HH-derived Daoy cells because of their non-MYC-amplified nature or low MYC copy number. Together, our results demonstrate that the combination of BEZ235 and Vismodegib does target the PI3K-mTOR and HH signaling pathways, and thereby decreases cell growth and induces apoptosis in SHH/MYC-driven MB cells.

In addition to the pharmacological inhibition of these target pathways, we performed a genetic knock-down experiment using siRNA approach to inhibit mTOR/S6K pathway in MB cells. Our results showed that knocked-down of MB cells with S6K-siRNA significantly inhibited cell growth by downregulating the expression levels of GLI1 and MYC proteins in HH- and MYC-driven MB cells, respectively (Supplementary Figure 3), suggesting that mTOR-S6K1 cooperates with the SHH pathway and MYC activity leading to enhanced MB tumorigenesis and resistance. These molecular analyses further support the hypothesis that there is crosstalk between the mTOR translation pathway and HH/MYC.

### Inhibitors chemosensitize MB cells

**Figure 4:**
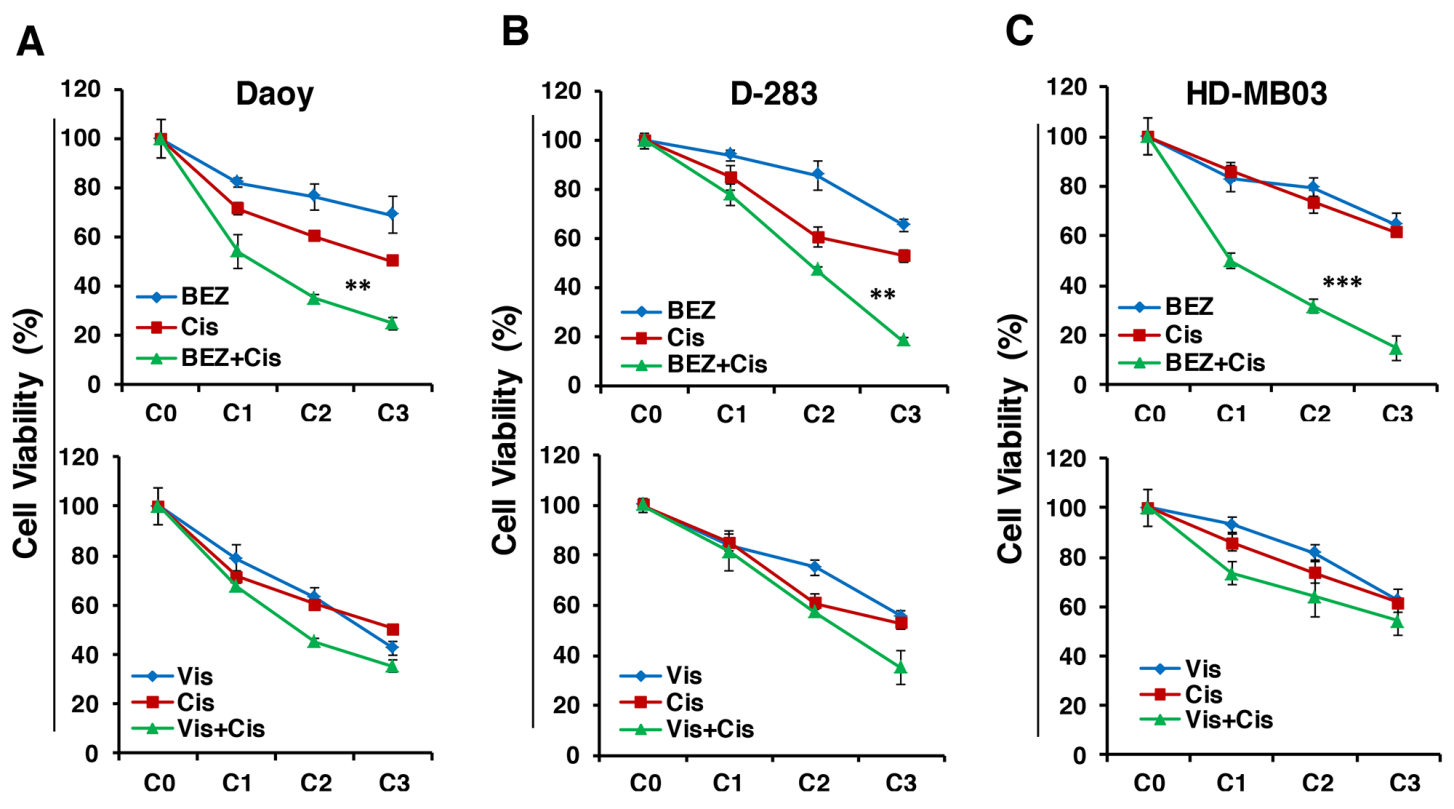
Effects of inhibitors in combination with chemotherapy on MB cell growth MTT results showing the effects of Vis or BEZ individually or combined with Cis in Daoy **(A)**, D-283 **(B)** and HD-MB03 **(C)** MB cell lines for 72 hours in a dose-dependent manner. The inhibitors concentrations C0, C1, C2 and C3 indicate increasing 0, 5, 10 and 20 μM/nM concentrations of Vis and BEZ, respectively. For Cis, C0, C1, C2 and C3 indicate their 0, 0.5, 1 and 1.5 μM concentrations. The values represent the means ± SD from four wells of 96-well plates. ^**^p<0.01; ^***^p<001.

Given the limited success of current therapies, we next sought to determine whether Vismodegib or BEZ235 could enhance the anti-MB efficacy of chemotherapy by sensitizing MB cells. Cisplatin is being used as a first line chemotherapeutic drug in the treatment of MB [39]. To evaluate the enhanced efficacy of inhibitors on cisplatin-mediated MB cytotoxicity, we treated MB (Daoy, D-283 and HD-MB03) cells with inhibitors and cisplatin alone or combined, in a dose-dependent fashion for 72 h and determined cell growth using MTT assay. Results shown in Figure 4, clearly indicate that co-treatment of MB cells with inhibitors (BEZ235 or Vismodegib) and cisplatin significantly inhibited cell growth in a dose-dependent manner. Of these combinations, BEZ235 demonstrated a significantly greater efficacy in enhancing cisplatin-mediated MB cytotoxicity. Results also indicated a higher sensitivity of MYC-amplified MB cells to these combined treatments compared to non-MYC-amplified cells (Figure 4). Our combination index (CI) analyses between inhibitors and cisplatin in MB cell lines confirmed that there was strong synergy between BEZ235 and cisplatin with CI ~0.3-0.8. However, Vismodegib combined with cisplatin showed the additive interaction with CI ranges 0.81-1.1 (Table 1). These data further confirmed that the combination of BEZ235 and cisplatin was most efficacious against MB.

**Figure 5:**
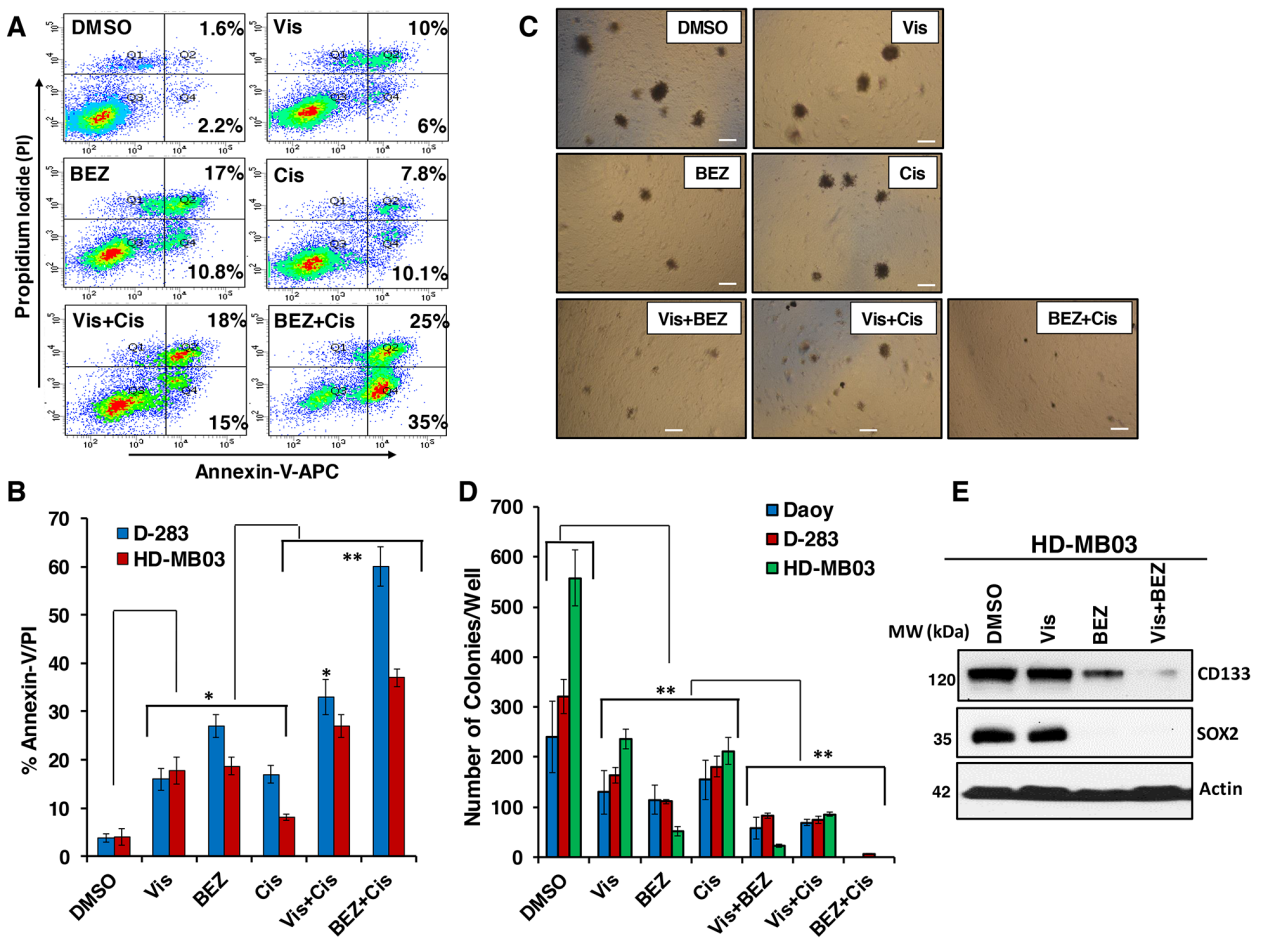
Effects of inhibitors in combination with chemotherapy on MB apoptosis and colony formation **(A)** Shows a representative scatter diagram for the apoptotic cell analyses following treatment with vehicle (DMSO) or Vis 20 μM, BEZ 20 nM and Cis 1 μM alone or combined as indicated in MB cells. **(B)** Quantification of the apoptotic cells (% Annexin-V/PI) following inhibitors alone or in combination with cisplatin treatment in D-283 and HD-MB03 MB cells. The values represent the means ± SD of three separate experiments. ^*^, p<0.05; ^**^, p<0.01. **(C)** Shows a representative micrograph for the colony forming ability of MB cells with the treatments of vehicle or Vis (20 μM), BEZ (20 nM) and Cis (1 μM) alone or combined for 15 days. The micrographs were taken using a phase contrast microscope at 10x magnification. Scale bar; 100 μm. **(D)** Quantification of the number of colonies following treatment with inhibitors alone or combined in Daoy, D-283 and HD-MB03 MB cells. The values represent the means ± SD from three wells of 6-well plates. ^*^p<0.01. **(E)** Western blot analyses for the expression levels of CD133 and SOX2 following the indicated treatments of HD-MB03 spheres for 48 hours. β-Actin was used as a loading control in this experiment.

To further test the effects of these combinations on MB apoptosis, we determined Annexin-V/PI double positive cells using flow cytometric analysis. The results of these analyses showed significantly increased apoptosis by inhibitors alone (Figure 5A and 5B). In combination with cisplatin, inhibitors further significantly increased the apoptosis in both HH/MYC-driven MB cell lines compared to the respective single agents (Figure 5B). The combination efficacy of these inhibitors in inducing apoptosis, were consistent with the growth inhibitory effects. Together, these results suggested that although inhibitors and cisplatin showed anti-MB efficacies alone, the combination treatments significantly sensitized MB cells to enhanced growth inhibition and apoptosis, in part mediated by targeting HH and PI3K-mTOR pathways.

### Combination efficacies of Inhibitors on MB colony formation

To further validate the effects of inhibitors on anchorage-independent growth (clonogenicity) in MB cells in an *in vitro assay* for tumorigenicity, we performed colony formation assay using semi-solid agar medium. Figure 5C shows a representative micrograph picture for colony forming ability in control, inhibitor alone and inhibitor combined-treated MB cells. We found that both inhibitors and cisplatin as single agents significantly decreased the numbers of colonies when compared to vehicle treated cells (Figure 5C and 5D). Interestingly, compared to their efficacy as single agents, inhibitors Vismodegib and BEZ235 combined together, or individually combined with cisplatin, induced a significant reduction in the number of colonies in all MB lines (Figure 5D), indicating potency of these inhibitors to inhibit colony formation/tumorigenicity. These results also showed that there was a much more marked inhibition in the colony formation capability by inhibitors compared to cell growth/proliferation (Figure 2 and 4). Consistent with earlier observations, BEZ235 efficacy, either alone or combined, was most efficacious in inhibiting colony forming ability of MB cells. We did not observe significant differences among MB cell lines in their response to therapy. However, the HD-MB03 cell line showed significantly higher colony forming ability compared to D-283 and D-341 MB lines, indicating the more aggressive behavior of HD-MB03 MB cells.

We further tested the combined effects of inhibitors Vismodegib and BEZ235 on expression levels of neural stem cell markers (CD133 and SOX2) in HD-MB03 MB spheres by western blotting. Results shown in Figure 5E demonstrated that as a single agent, BEZ235 was able to significantly inhibit the expression of both CD133 and SOX2. BEZ235 treatment resulted in complete shut-down of SOX2 expression in MB cells. However, we did not observe any significant effects of Vismodegib on the expression of these markers. The results also clearly demonstrated a significantly further decreased expression of CD133 when the inhibitors were combined. Together, these data suggested that combined inhibitors targeted the molecules associated with tumorigenic (cancer) stem cells thereby inhibiting colony formation.

### Combination efficacies of inhibitors *in vivo* in a xenograft mouse model

**Figure 6:**
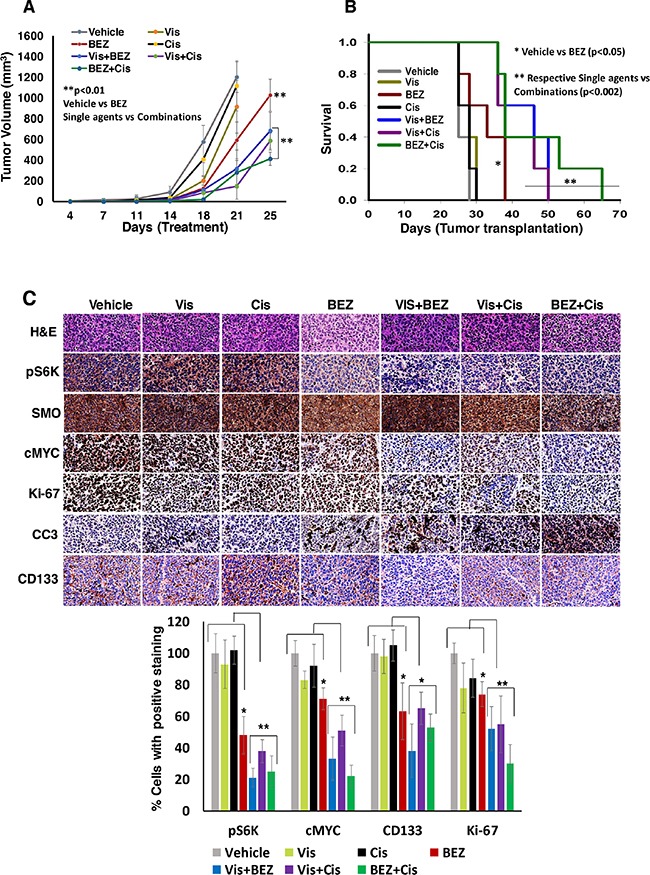
Combined *in vivo* anti-MB efficacies of inhibitors against HD-MB03 xenografts mice NSG mice bearing MYC-driven (HD-MB03) MB tumors were treated with vehicle or Vis (50 mg/kg), BEZ (25 mg/kg) and Cis (2 mg/kg) alone or combined as indicated, twice a week for four weeks. **(A)** Tumor growth analyses following treatments. **(B)** Kaplan–Meier analyses for the survival of mice using the log-rank test. **(C)** Histological (H&E) and corresponding immunohistochemical analyses (pS6K, SMO, cMYC, Ki-67, CD133 and cleaved caspase-3) of xenograft tumor tissue, as indicated. The bar graph shows the quantified expression levels of indicated proteins using tumor tissues of three xenograft mice from each treatment group. In these quantifications, immunostaining of vehicle-treated tumors was set as 100% staining for the comparison between the groups. ^*^p<0.05; ^**^p<0.01. The images were scanned and captured using digital scanner VENTANA Image software (Roche, Germany) at 40× magnification. The results shown in this figure represent one of the two independent experiments. In one experiment, we used 7 mice per group and in another experiment, 5 mice per group. There were no significant differences observed on anti-MB efficacies of inhibitors in these two experiments.

As a next logical step, to validate our *in vitro* results, we further tested the single agents and combined efficacies of inhibitors in NSG mice bearing aggressive MYC-amplified HD-MB03 MB cells. The tumor bearing mice were treated with inhibitors Vismodegib, BEZ235, cisplatin alone or their combinations. Results shown in Figure 6 show the single agent and combined efficacies of inhibitors on MB tumor growth and survival in NSG xenograft mice. As single agents, Vismodegib and cisplatin slightly decreased MB tumor growth compared to vehicle treatment, there were no statistically significant effects on tumor growth by these agents. However, BEZ235 as single agent, significantly (p<0.01) delayed tumor growth of NSG xenografts (Figure 6A). Expectedly, compared to their efficacy as single agents, Vismodegib and BEZ235 combined together, or individually combined with cisplatin significantly (p<0.01) delayed tumor growth over a 3-week period, suggesting that combinations have potency to inhibit MYC-driven MB proliferation *in vivo*. We next determined the survival of these treated mice. A maximum 2 cm^3^ tumor size was set as end point for the survival analyses. The survival data clearly demonstrated that xenografted mice treated with inhibitors Vismodegib and BEZ235 together or individually combined with cisplatin exhibited a highly significantly increased survival when compared to single agents-treated mice (Figure 6B) and these results were consistent with *in vivo* tumor growth studies. In addition, treatments with these inhibitors did not cause a significant reduction in the total body weights between control and treatment groups (See Supplementary Figure 4), suggesting the tolerability of these combinations.

Further, we determined the effect of inhibitors alone or combined on the expression of phospho-S6K (mTOR pathway), SMO (HH pathway) c-MYC (a hallmark in MYC-driven MB), Ki-67 (proliferation) and cleaved caspase3 (apoptosis) molecules in xenograft tumors. Results from immunohistochemical analyses (Figure 6C) showed that the combination of Vismodegib and BEZ235 together or individually combined with cisplatin significantly decreased the expression levels of phosphorylated S6K (mTOR), c-MYC, and Ki-67, and increased levels of cleaved (activated) caspase 3, suggesting the combinations not only enhanced reduction in tumor growth and increased survival, but also targeted relevant associated pathways and downstream molecules in the xenografted tumors. In addition, these combinations were able to inhibit the expression levels of neural stem cell marker CD133, indicating the potencies of these combinations to inhibit tumor stem cell survival or self-renewal of MB tumor cells *in vivo*.

## DISCUSSION

Current treatments can cure a majority of patients diagnosed with MB with the exception of some patients with high-risk tumors. However, these therapies are associated with significant long-term toxicities in children. Patients with high-risk disease, particularly HH/MYC-driven MB face a paucity of effective therapies [10]. MB represents a diverse and genetically heterogeneous group of tumors, which makes the implementation of molecular therapies challenging [9, 10]. Recently, there have been promising genomic and transcriptional profiling studies in MB that suggest that this disease has distinct molecular subgroups, some of which may be amenable to targeted therapy [3, 9]. Molecular and preclinical studies have revealed the aberrant activation and crosstalk between HH and PI3K-mTOR signaling pathways leading to tumorigenesis and drug-resistance in both HH- and MYC-driven MB [13, 14, 18, 23, 25]. Therefore, the combined targeting of these oncogenic pathways using small molecule inhibitors that have molecular specificity and enhance cytotoxic chemotherapy may provide improved therapy in high-risk MB without major toxicities. Here, we show that combined targeting of HH and PI3K-mTOR signaling pathways using small molecule inhibitors, exhibited broad anti-tumor effects against MB *in vitro* and *in vivo*.

Since HH- and MYC-driven molecular subgroups frequently represent a high-risk disease in MB patients [1], we used a HH-driven Daoy (non-MYC amplified) MB cell line and three MYC-driven/amplified (D-283, D-341 and HD-MB03) MB cell lines [37] in the present study. Our findings with single agent activity of the inhibitors, using these cell lines *in vitro*, indicated superior efficacy of BEZ235 compared to Vismodegib activity, suggesting the role of PI3K-mTOR pathway in both HH- and MYC-driven MB tumorigenesis. Our results also demonstrated that MYC-amplified cells show greater sensitivity to PI3K-mTOR inhibitor and lower sensitivity toward chemotherapy as IC_50_ values shifted lower for BEZ235 and higher for cisplatin in these cells compared to non-MYC-amplified cells, indicating the higher sensitivity of MYC-amplified MB cells to BEZ235 and the relatively chemoresistant nature of these cells.

Although our studies demonstrate that Vismodegib can specifically target SHH signaling and inhibit MB cell growth, it showed limited efficacies with high IC_50s_ against HH/MYC-driven MB cells. However, it is evident from primary MB and preclinical mouse model studies that the SMO (HH) inhibitors including Vismodegib are not effective in SHH/MYC-driven MB tumors due to mutation/amplification of HH signaling components and abnormal activation of PI3K-mTOR signaling [25, 40], suggesting that these abnormalities might have role in making Vismodegib less effective and promoting resistant to therapy. It is also evident that non-canonical (SMO-independent) regulation of HH signaling by certain oncogenic signaling pathways such as PI3K-AKT-mTOR and RAS/MAPK can regulate HH downstream components such as the GLI transcription factor family [14–16]. Recently, inhibiting downstream target GLI transcription factor has emerged as a more promising therapy for various cancers including MB [41]. In addition, the FDA-approved GLI1/GLI2 inhibitor Arsenic-trioxide (ATO), is currently under phase I/IV clinical trials for various solid and hematological malignancies [41, 42] and therefore, warrants further evaluation in combination with other key signaling pathways inhibitors for therapy of the HH-driven tumors such as MB.

Our *in vitro* findings clearly demonstrated that combined targeting of PI3K-mTOR and HH signaling pathways significantly inhibits proliferation and survival in both HH- and MYC-driven MB cells. Molecularly, combined inhibition of these pathways, significantly downregulated the expression of HH and PI3K-mTOR key signaling components and downstream targets. These observations indicate crosstalk between PI3K-mTOR and HH signaling pathways in aggressive MBs. Based on our analyses of the results, the significant increase in therapeutic efficacy of the combination of these two inhibitors appears due to synergistic effects. Overall, our molecular analyses of these inhibitors demonstrate their efficacy in inhibiting MB cell growth and improving survival by targeting associated pathways and their regulated downstream molecules. Since both HH and PI3K-mTOR signaling pathways are frequently associated with chemoresistance in various cancers including MB [5, 18], we also sought to see whether inhibition of these signaling pathways contributes to chemotherapy-enhanced activity against MB. Our results revealed that both inhibitors significantly enhanced the cisplatin-induced growth inhibition and apoptosis in MB cells, suggesting that inhibition of HH and PI3K-mTOR pathways not only suppresses cell proliferation and survival of MB cells, but also chemosensitize MB cells.

Both HH and PI3K-mTOR pathways have been implicated as having roles in cancer “stem” cells and tumor-initiating cells, leading to tumor relapse and drug-resistance in various malignancies including MB [11, 43]. MB cells express neural stem cell markers such as CD133, SOX2 and Nestin and have ability to form colonies/spheres [44, 45]. Our results provide evidence that the inhibitors as single agents, or combined, reduced colony forming abilities of MB cells. Consistent with earlier findings, BEZ235 efficacy, either alone or combined, was most efficacious in inhibiting colony forming abilities. Also, among cell lines, a MYC-driven HD-MB03 cell line showed a significantly higher colony forming ability compared to other MB lines and indicated a more aggressive behavior of HD-MB03 MB cells. Our results for the combination efficacy of Vismodegib and BEZ235 provide evidence that the combination significantly downregulated the expression of the neural stem cell markers CD133 and SOX2 in aggressive HD-MB03 MB cells. Although Vismodegib alone did not alter the expression of these stem cell markers, its combination with BEZ235, led to a reduction in the expression of these markers. This indicates the importance of combination therapy for the targeting of “cancer stem cells”. These results suggest the impact of inhibitors on tumor cell self-renewal, with the potential of reducing recurrence of MB, thus improving progression-free survival.

Having shown the *in vitro* effects of our combined approach, as a next logical step, we investigated the anti-MB efficacy of combined approaches *in vivo* using NSG mice bearing the most aggressive MYC-driven HD-MB03 MB tumors. Our results from this study indicate that as single agent, only BEZ235 effectively delayed tumor growth and increased survival in xenografted mice. However, the combinations of both Vismodegib and BEZ235 together or individually combined with cisplatin significantly delayed tumor growth and increased survival in mice compared to respective single agent activity, suggesting anti-MB combination potency and chemotherapy-enhancing efficacy of these inhibitors *in vivo*. In addition, the combination of agents as described above, significantly downregulated the expression levels of key components in HH/PI3K-mTOR signaling pathway including downstream targets, suggesting the combinations not only reduced tumor growth, but also targeted associated pathways and downstream molecules in xenograft tumors. As a single agent, we did not observe significant anti-MB efficacy of Vismodegib and cisplatin on tumor growth and/or survival, suggesting MYC-driven MB resistance to this HH inhibitor and chemotherapy which is consistent with our *in vitro* studies. However, these agents exhibited anti-MB efficacies when combined with BEZ235, suggesting the role of the PI3K-mTOR pathway in HH-resistance and MYC-driven MB progression. Together, the data from the *in vivo* studies further confirm and support our hypothesis of a critical role of the mTOR translation pathway in HH/MYC- driven MB.

Both Vismodegib and BEZ235 can cross the blood-brain barrier, making it an attractive option for the treatment of MB [46, 47]. Both subcutaneous and orthotopic xenograft models have been used in MB studies. Subcutaneous models are suitable for initial drug testing and screening as this model allows for easy tumor visualization and quantification, making decisions of treatment initiation and drug application less difficult [48]. Orthotopic/intracranial models of MB require additional features such as modification of MB cells in order to image and quantitate tumor growth, or modification of clinical imaging approaches e.g. MRI, CT/SPECT. Patient derived xenograft and orthotopic models for MB and clinically relevant imaging are currently under development in our laboratory for future animal studies.

In summary, combinations of both HH and PI3K-mTOR signaling pathway inhibitors together, or with chemotherapy, demonstrated significant preclinical efficacy in reducing MB cell growth, inducing apoptosis with prolongation of survival in a xenograft model. In these combinations, the PI3K-mTOR dual-inhibitor BEZ235 combined with chemotherapy showed the greatest increased efficacy both *in vitro* and *in vivo*. The findings from this study warrants further preclinical evaluation in patient-derived tumor xenografts and clinical investigation, in order to translate these approaches to the clinical setting, to determine their efficacies in high-risk MB patients.

## MATERIALS AND METHODS

### Cell lines and maintenance

The human MB cell lines Daoy, D-283 and D-341and were obtained from American Type Culture Collection (ATCC, Manassas, VA). HD-MB03 MB cell line was obtained from Deutsche Sammlung von Mikroorganismen und Zellkulturen (DSMZ, Germany). The cell lines were authenticated by their respective supplier. These cell lines were cultured in EMEM media (ATCC) containing 10% FBS, 1% penicillin, and 1% streptomycin (Invitrogen, CA). The cultures were maintained in a humidified incubator at 5% CO2 and 95% air atmosphere at 37°C. All cultures were passaged at 80-90% confluence.

### The therapeutic agents

The HH inhibitor Vismodegib and PI3K-mTOR dual inhibitor BEZ235 were purchased from LC laboratories (Woburn, MA) and a chemotherapeutic drug, cisplatin was purchased from Selleckchem Company (Houston TX). These inhibitors were dissolved in DMSO at appropriate concentrations and stored at −20°C.

### *In vitro* growth assay

To determine therapeutic efficacies of single and combined inhibitors, twenty thousand cells of each MB line were plated and treated with inhibitors in a dose-dependent fashion in 96-well plates and the growth of these cells was determined at 72 hours using an MTT assay. The IC_50_ values of each drug for each cell line were determined using GraphPad Prism V6 software. Briefly, 25 μl of MTT reagent (5 mg/ml in PBS) was added to the cultures and incubated for 2 hours before the respective time point, and the cells were lysed using an SDS-based lysing reagent. The intensity of the color developed was determined at a 570 nm wavelength using a plate reader (Biotek, Germany).

### Colony forming assay

To determine the efficacy of inhibitors on colony forming ability of MB cells, colony forming assays were performed using 0.3% agar semi-solid medium. One-hundred thousand cells from each cell line were mixed with the aforementioned medium, containing DMSO or inhibitors alone/or combined and plated in triplicate in 6-well plates and incubated at 37°C with 5% CO_2_ for 2 weeks. Following incubation, aggregates of cells with >50 μm size were counted as colonies using an inverted microscope. The MB cells were also grown as spheres in low-adherent 12-well plates using neural stem cell (serum-free) media and treated with inhibitors for 48 hours. Following treatments, sphere lysate was prepared and subjected to western blot analysis for the expression of neural stem cell markers.

### Apoptosis assay

The ability of inhibitors to induce apoptosis in MB cell lines was determined using an Annexin-V:APC flow cytometry assay kit (BD Biosciences, CA) following the manufacturer's instructions. Briefly, 0.3 × 10^6^ cells/ml MB cell lines were plated in 12-well plates and treated with inhibitors alone or in combination for 72 hours. The percent of the cells undergoing apoptosis was then assessed using Annexin-V/propidium iodide double staining. In some experiments, the induction of apoptosis by inhibitors was also determined using Caspase 3/7 activity assay kit (Promega, WI) following the manufacturer's instructions.

### Western blotting

Western blot analysis of the inhibitor-treated cells was performed using a standardized protocol [35]. The primary human antibodies used in these analyses included c-MYC, SMO, and β-Actin (Santacruz, CA), AKT, phospho-AKT, S6K, phospho-S6K, GLI1 and SOX2 (Cell Signaling Technology, MA) and, cyclin D1, Bcl-2 and CD133 (BD Biosciences, CA). Immunoreactivity was detected using appropriate peroxidase-conjugated secondary antibodies (Santacruz, CA) and visualized using an enhanced chemiluminescence (ECL) detection system (Pierce, IL).

### Quantitative RT-PCR (qRT-PCR)

Total RNA was prepared by using Trizol reagent (Invitrogen, CA) and 2 μg of total RNA was used for cDNA preparation using superscript verso enzyme kit (Promega). cDNA product was amplified in 10 μl reaction and amplified using SYBR Green Super Mix (Applied Biosystems) and GLI1 gene specific (Forward: 5′-CCAACTCCACAGGCATACAGGAT-3; Reverse: 5′-CACAGATTCAGGCTCACGCTTC-3′) primers. All reactions were processed in QuantStudio 3 Real-Time PCR System and results were analyzed by data analysis software (Applied Biosystems) by normalizing with housekeeping gene GAPDH level.

### siRNA knock-down experiment

Control (Scrambled) and S6K1 siRNAs were purchased from Santacruz Biotechnology CA. siRNAs were transfected in MB cells using Lipofectamine 2000 (Invitrogen, CA) following manufacturer's instructions. Following 48 hours of transfections, cells were subjected to western blotting for the expression of proteins as mentioned and MTT assay for the cell growth analysis.

### *In vivo* studies

All animal experiments were performed according to a UNMC Institutional Animal Care and Use Committee (IACUC) approved protocol. For these studies, six- to eight-week-old NOD-SCID common gamma chain knockout (NSG) mice from Jackson Laboratories (Bar Harbor, ME) were injected subcutaneously in the flank with 2.5 × 10^5^ HD-MB03 MB cells suspended in 100 μl PBS and mixed 1:1 with matrigel (BD Biosciences). Ten days post-tumor injection, when tumor was palpable, the tumor bearing mice were divided into seven treatment groups (n=6 per group) and treated twice a week for four weeks. Treatments included vehicle control (NMP/polyethylene-glycol-300 at 10/90, v/v ratio, i.p.), Vismodegib (50 mg/kg, i.p.), BEZ235 (25 mg/kg, i.p.), cisplatin (2 mg/kg, i.p.), Vismodegib (50 mg/kg)+BEZ235 (25 mg/kg), Vismodegib (50 mg/kg)+cisplatin (2 mg/kg) and BEZ235 (25mg/kg)+cisplatin (2mg/kg). The doses used for these inhibitors were at ranges of achievable exposures in mice or humans [32–34, 36]. Tumor growth was assessed twice a week using a digital caliper and tumor volume was quantitated using the formula (length x width^2^ x 0.5). When tumor volume approached 2 cm^3^, the mice were euthanized using CO_2_ and tumor tissues were collected and processed for the histological/immunohistological analyses. The survival of the vehicle or different agent–treated mice was determined by the Kaplan–Meier method and analyzed for statistical significance using the log rank test.

### Statistical analysis

Each experiment was performed in triplicate and repeated an additional 2–3 times and the mean and standard error values of all experiments calculated. The significance of differences (p-value) was calculated using independent Student t-tests and p-values less than 0.05 considered significant. To determine inhibitor combinations/interactions, we used the Chou and Talalay CI method using CalcuSyn software (Biosoft, Cambridge, UK). CI<0.9 indicates synergism, 0.9-1.1 additivity and >1.1 antagonism.

## SUPPLEMENTARY MATERIALS FIGURES



## References

[1] Gajjar AJ, Robinson GW (2014). Medulloblastoma-translating discoveries from the bench to the bedside. Nat Rev Clin Oncol.

[2] Taylor MD, Northcott PA, Korshunov A, Remke M, Cho YJ, Clifford SC, Eberhart CG, Parsons DW, Rutkowski S, Gajjar A, Ellison DW, Lichter P, Gilbertson RJ (2012). Molecular subgroups of medulloblastoma: the current consensus. Acta Neuropathol.

[3] Jones DT, Jäger N, Kool M, Zichner T, Hutter B, Sultan M, Cho YJ, Pugh TJ, Hovestadt V, Stütz AM, Rausch T, Warnatz HJ, Ryzhova M (2012). Dissecting the genomic complexity underlying medulloblastoma. Nature.

[4] Ng JM, Curran T (2011). The Hedgehog's tale: developing strategies for targeting cancer. Nat Rev Cancer.

[5] Huang SY, Yang JY (2015). Targeting the Hedgehog pathway in pediatric medulloblastoma. Cancers (Basel).

[6] Ransohoff KJ, Sarin KY, Tang JY (2015). Smoothened inhibitors in sonic hedgehog subgroup medulloblastoma. J Clin Oncol.

[7] Yauch RL, Dijkgraaf GJ, Alicke B, Januario T, Ahn CP, Holcomb T, Pujara K, Stinson J, Callahan CA, Tang T, Bazan JF, Kan Z, Seshagiri S (2009). Smoothened mutation confers resistance to a Hedgehog pathway inhibitor in medulloblastoma. Science.

[8] Dijkgraaf GJ, Alicke B, Weinmann L, Januario T, West K, Modrusan Z, Burdick D, Goldsmith R, Robarge K, Sutherlin D, Scales SJ, Gould SE, Yauch RL, de Sauvage FJ (2011). Small molecule inhibition of GDC-0449 refractory smoothened mutants and downstream mechanisms of drug resistance. Cancer Res.

[9] DeSouza RM, Jones BR, Lowis SP, Kurian KM (2014). Pediatric medulloblastoma - update on molecular classification driving targeted therapies. Front Oncol.

[10] MacDonald TJ, Aguilera D, Castellino RC (2014). The rationale for targeted therapies in medulloblastoma. Neuro Oncol.

[11] Justilien V, Fields AP (2015). Molecular pathways: novel approaches for improved therapeutic targeting of Hedgehog signaling in cancer stem cells. Clin Cancer Res.

[12] Di Magno L, Coni S, Di Marcotullio L, Canettieri G (2015). Digging a hole under Hedgehog: downstream inhibition as an emerging anticancer strategy. Biochim Biophys Acta.

[13] D'Amico D, Canettieri G (2016). Translating hedgehog in cancer: controlling protein synthesis. Trends Mol Med.

[14] Brechbiel J, Miller-Moslin K, Adjei AA (2014). Crosstalk between hedgehog and other signaling pathways as a basis for combination therapies in cancer. Cancer Treat Rev.

[15] Wang Y, Ding Q, Yen CJ, Xia W, Izzo JG, Lang JY, Li CW, Hsu JL, Miller SA, Wang X, Lee DF, Hsu JM, Huo L (2012). The crosstalk of mTOR/S6K1 and Hedgehog pathways. Cancer Cell.

[16] Zhao X, Ponomaryov T, Ornell KJ, Zhou P, Dabral SK, Pak E, Li W, Atwood SX, Whitson RJ, Chang AL, Li J, Oro AE, Chan JA (2015). RAS/MAPK activation drives resistance to SMO inhibition, metastasis, and tumor evolution in Shh pathway-dependent tumors. Cancer Res.

[17] Metcalfe C, Alicke B, Crow A, Lamoureux M, Dijkgraaf GJ, Peale F, Gould SE, de Sauvage FJ (2013). PTEN loss mitigates the response of medulloblastoma to Hedgehog pathway inhibition. Cancer Res.

[18] Dimitrova V, Arcaro A (2015). Targeting the PI3K/AKT/mTOR signaling pathway in medulloblastoma. Curr Mol Med.

[19] Mainwaring LA, Kenney AM (2011). Divergent functions for eIF4E and S6 kinase by sonic hedgehog mitogenic signaling in the developing cerebellum. Oncogene.

[20] Eckerdt F, Beauchamp E, Bell J, Iqbal A, Su B, Fukunaga R, Lulla RR, Goldman S, Platanias LC (2014). Regulatory effects of a Mnk2-eIF4E feedback loop during mTORC1 targeting of human medulloblastoma cells. Oncotarget.

[21] Pambid MR, Berns R, Adomat HH, Hu K, Triscott J, Maurer N, Zisman N, Ramaswamy V, Hawkins CE, Taylor MD, Dunham C, Guns E, Dunn SE (2014). Overcoming resistance to Sonic Hedgehog inhibition by targeting p90 ribosomal S6 kinase in pediatric medulloblastoma. Pediatr Blood Cancer.

[22] Cage TA, Chanthery Y, Chesler L, Grimmer M, Knight Z, Shokat K, Weiss WA, Gustafson WC (2015). Downregulation of MYCN through PI3K inhibition in mouse models of pediatric neural cancer. Front Oncol.

[23] Buonamici S, Williams J, Morrissey M, Wang A, Guo R, Vattay A, Hsiao K, Yuan J, Green J, Ospina B, Yu Q, Ostrom L, Fordjour P (2010). Interfering with resistance to smoothened antagonists by inhibition of the PI3K pathway in medulloblastoma. Sci Transl Med.

[24] Eckerdt F, Goldman S, Platanias LC (2014). New insights into malignant cell survival mechanisms in medulloblastoma. Cancer Cell Microenviron.

[25] Pei Y, Moore CE, Wang J, Tewari AK, Eroshkin A, Cho YJ, Witt H, Korshunov A, Read TA, Sun JL, Schmitt EM, Miller CR, Buckley AF (2012). An animal model of MYC-driven medulloblastoma. Cancer Cell.

[26] Kawauchi D, Robinson G, Uziel T, Gibson P, Rehg J, Gao C, Finkelstein D, Qu C, Pounds S, Ellison DW, Gilbertson RJ, Roussel MF (2012). A mouse model of the most aggressive subgroup of human medulloblastoma. Cancer Cell.

[27] Hill RM, Kuijper S, Lindsey JC, Petrie K, Schwalbe EC, Barker K, Boult JK, Williamson D, Ahmad Z, Hallsworth A, Ryan SL, Poon E, Robinson SP (2015). Combined MYC and P53 defects emerge at medulloblastoma relapse and define rapidly progressive, therapeutically targetable disease. Cancer Cell.

[28] Staal JA, Pei Y, Rood BR (2016). A proteogenomic approach to understanding MYC function in metastatic medulloblastoma tumors. Int J Mol Sci.

[29] Pei Y, Liu KW, Wang J, Garancher A, Tao R, Esparza LA, Maier DL, Udaka YT, Murad N, Morrissy S, Seker-Cin H, Brabetz S, Qi L (2016). HDAC and PI3K antagonists cooperate to inhibit growth of MYC-driven medulloblastoma. Cancer Cell.

[30] Moavero R, Folgiero V, Carai A, Miele E, Ferretti E, Po A, Diomedi Camassei F, Lepri FR, Vigevano F, Curatolo P, Valeriani M, Colafati GS, Locatelli F (2016). Metastatic group 3 medulloblastoma in a patient with tuberous sclerosis complex: case description and molecular characterization of the tumor. Pediatr Blood Cancer.

[31] Roussel MF, Robinson GW (2013). Role of MYC in Medulloblastoma. Cold Spring Harb Perspect Med.

[32] Wong H, Alicke B, West KA, Pacheco P, La H, Januario T, Yauch RL, de Sauvage FJ, Gould SE (2011). Pharmacokinetic-pharmacodynamic analysis of vismodegib in preclinical models of mutational and ligand-dependent Hedgehog pathway activation. Clin Cancer Res.

[33] Maira SM, Stauffer F, Brueggen J, Furet P, Schnell C, Fritsch C, Brachmann S, Chène P, De Pover A, Schoemaker K, Fabbro D, Gabriel D, Simonen M (2008). Identification and characterization of NVP-BEZ235, a new orally available dual phosphatidylinositol 3-kinase/mammalian target of rapamycin inhibitor with potent in vivo antitumor activity. Mol Cancer Ther.

[34] Robinson GW, Orr BA, Wu G, Gururangan S, Lin T, Qaddoumi I, Packer RJ, Goldman S, Prados MD, Desjardins A, Chintagumpala M, Takebe N, Kaste SC (2015). Vismodegib exerts targeted efficacy against recurrent Sonic Hedgehog-subgroup medulloblastoma: results from phase II Pediatric Brain Tumor Consortium Studies PBTC-025B and PBTC-032. J Clin Oncol.

[35] Chaturvedi NK, Rajule RN, Shukla A, Radhakrishnan P, Todd GL, Natarajan A, Vose JM, Joshi SS (2013). Novel treatment for mantle cell lymphoma including therapy-resistant tumor by NF-κB and mTOR dual-targeting approach. Mol Cancer Ther.

[36] Chaturvedi NK, McGuire TR, Coulter DW, Shukla A, McIntyre EM, Sharp JG, Joshi SS (2016). Improved therapy for neuroblastoma using a combination approach: superior efficacy with vismodegib and topotecan. Oncotarget.

[37] Ivanov DP, Coyle B, Walker DA, Grabowska AM (2016). *In vitro* models of medulloblastoma: choosing the right tool for the job. J Biotechnol.

[38] Ecker J, Oehme I, Mazitschek R, Korshunov A, Kool M, Hielscher T, Kiss J, Selt F, Konrad C, Lodrini M, Deubzer HE, von Deimling A, Kulozik AE (2015). Targeting class I histone deacetylase 2 in MYC amplified group 3 medulloblastoma. Acta Neuropathol Commun.

[39] Smith RL, Shi X, Estlin EJ (2012). Chemotherapy dose-intensity and survival for childhood medulloblastoma. Anticancer Res.

[40] Kool M, Jones DT, Jäger N, Northcott PA, Pugh TJ, Hovestadt V, Piro RM, Esparza LA, Markant SL, Remke M, Milde T, Bourdeaut F, Ryzhova M, ICGC PedBrain Tumor Project (2014). Genome sequencing of SHH medulloblastoma predicts genotype-related response to Smoothened inhibition. Cancer Cell.

[41] Infante P, Alfonsi R, Botta B, Mori M, Di Marcotullio L (2015). Targeting GLI factors to inhibit the Hedgehog pathway. Trends Pharmacol Sci.

[42] Beauchamp EM, Ringer L, Bulut G, Sajwan KP, Hall MD, Lee YC, Peaceman D, Ozdemirli M, Rodriguez O, Macdonald TJ, Albanese C, Toretsky JA, Uren A (2011). Arsenic trioxide inhibits human cancer cell growth and tumor development in mice by blocking Hedgehog/GLI pathway. J Clin Invest.

[43] Xia P, Xu XY (2015). PI3K/Akt/mTOR signaling pathway in cancer stem cells: from basic research to clinical application. Am J Cancer Res.

[44] Huang GH, Xu QF, Cui YH, Li N, Bian XW, Lv SQ (2016). Medulloblastoma stem cells: promising targets in medulloblastoma therapy. Cancer Sci.

[45] Zanini C, Ercole E, Mandili G, Salaroli R, Poli A, Renna C, Papa V, Cenacchi G, Forni M (2013). Medullospheres from DAOY, UW228 and ONS-76 cells: increased stem cell population and proteomic modifications. PLoS One.

[46] Paw I, Carpenter RC, Watabe K, Debinski W, Lo HW (2015). Mechanisms regulating glioma invasion. Cancer Lett.

[47] Gil del Alcazar CR, Hardebeck MC, Mukherjee B, Tomimatsu N, Gao X, Yan J, Xie XJ, Bachoo R, Li L, Habib AA, Burma S (2014). Inhibition of DNA double-strand break repair by the dual PI3K/mTOR inhibitor NVP-BEZ235 as a strategy for radiosensitization of glioblastoma. Clin Cancer Res.

[48] Fomchenko EI, Holland EC (2006). Mouse models of brain tumors and their applications in preclinical trials. Clin Cancer Res.

