# Supplementary material for: Improved therapy for medulloblastoma: targeting hedgehog and PI3K-mTOR signaling pathways in combination with chemotherapy
Source: Oncotarget. 2018 Mar 30;9(24):16619–33. doi: 10.18632/oncotarget.24618 (PMC5908274; doi:10.18632/oncotarget.24618)
Supplement: Supplementary file 1 [file oncotarget-09-16619-s001.pdf]

Improved therapy for medulloblastoma: targeting hedgehog and PI3K-mTOR signaling pathways in combination with chemotherapy

SUPPLEMENTARY MATERIALS

A

Vis IC_{50} (μM)

	Cell growth inhibition	GLI1 inhibition
Daoy	52	28.6
HD-MB03	84	>80

B

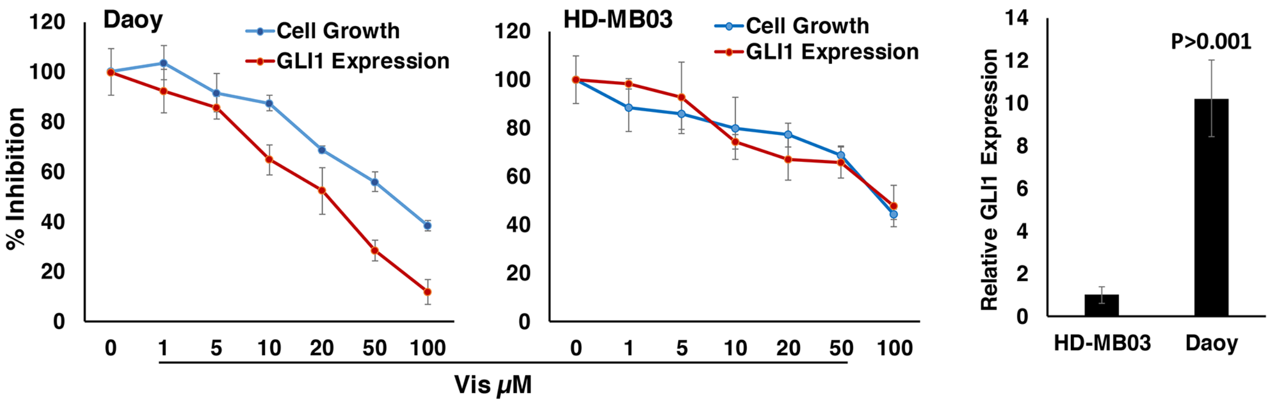

Supplementary Figure 1: Effect of Vismodegib (Vis) on MB cell growth and GLI1 expression. (A) MB cells were treated with Vis as indicated for 72 h. Following treatments, MB cells were subjected to cell growth analysis using MTT assay and GLI1 mRNA expression analysis using qPCR. The IC_{50} of Vis on MB cell growth and GLI1 expression were calculated using Prism version 6 software. **(B)** Relative expression of GLI1 between Daoy (SHH-driven) and HD-MB03 (MYC-driven) MB cell lines using qPCR.

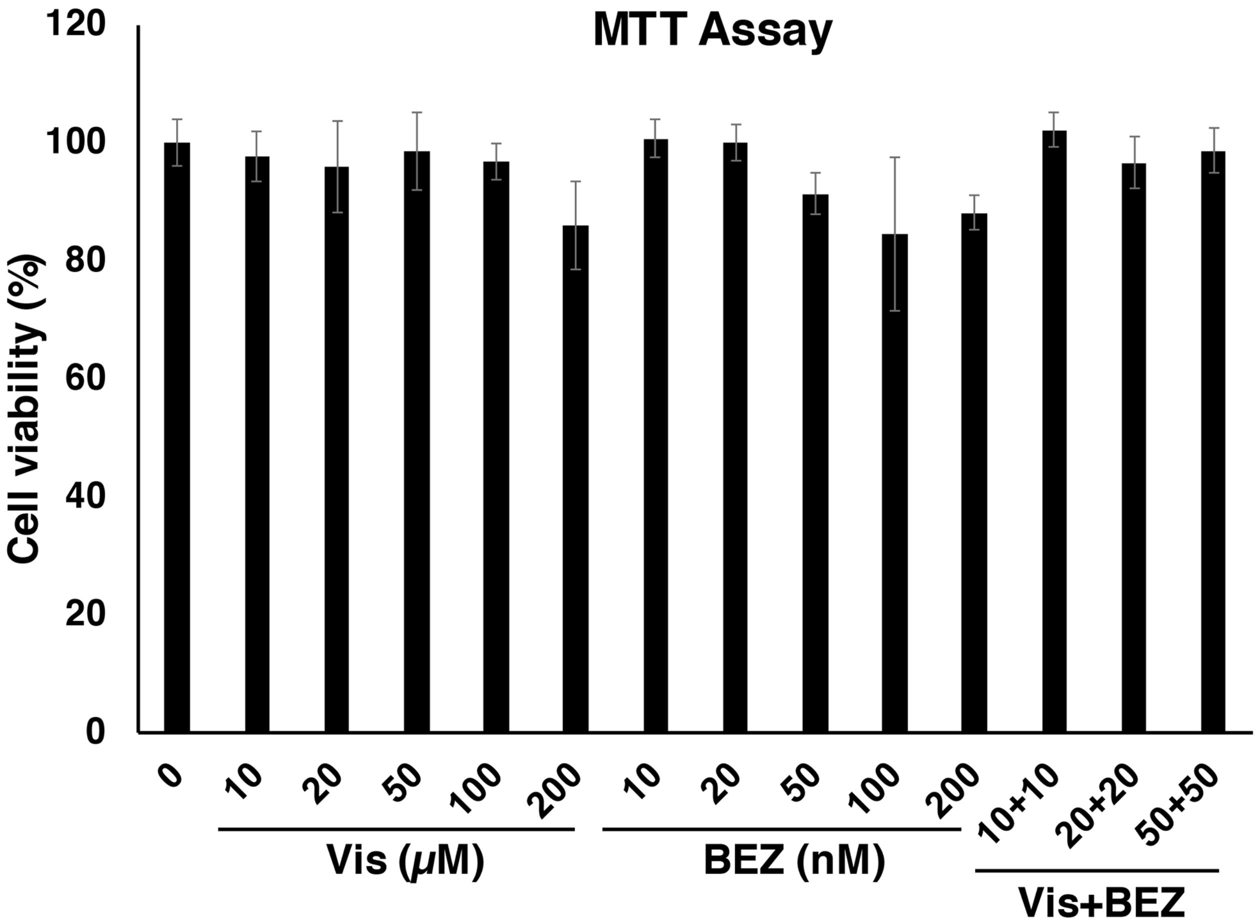

Supplementary Figure 2: Effects of inhibitors on the viability of normal peripheral blood mononuclear cells (PBMCs). PBMCs isolated from healthy donors were treated with Vismodegib (Vis) and BEZ235 (BEZ) alone or combined, as indicated for 72 h. Following treatments, MB cells were subjected to cell growth analysis using MTT assay.

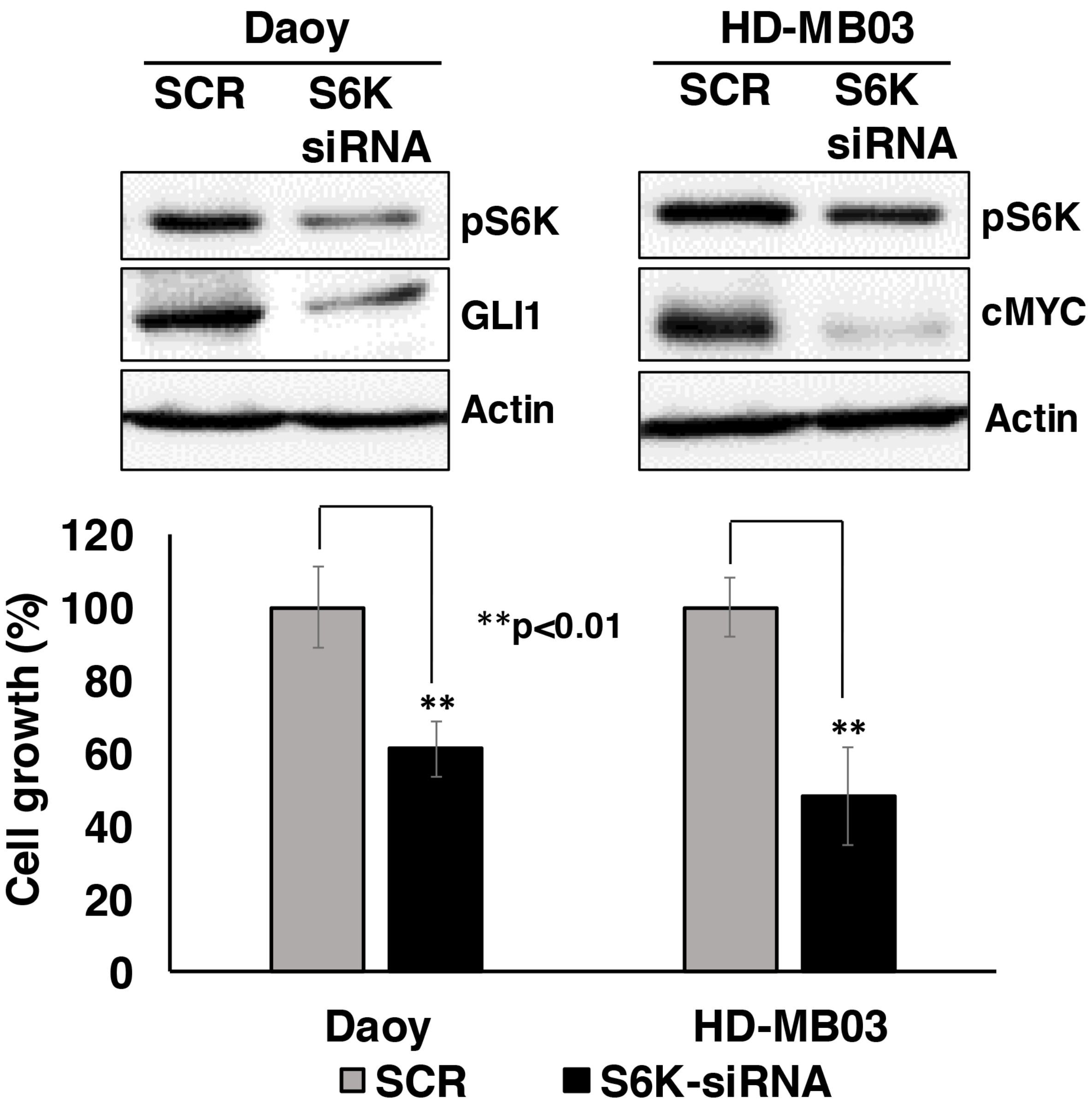

Supplementary Figure 3: Genetic inhibition of S6K in MB cells. Daoy and HD-MB03 cells were transiently transfected with control (SCR) and S6K siRNAs for 48 hours and were subjected to western blotting for the expression of indicated proteins and cell growth (viability) analysis using MTT assay.

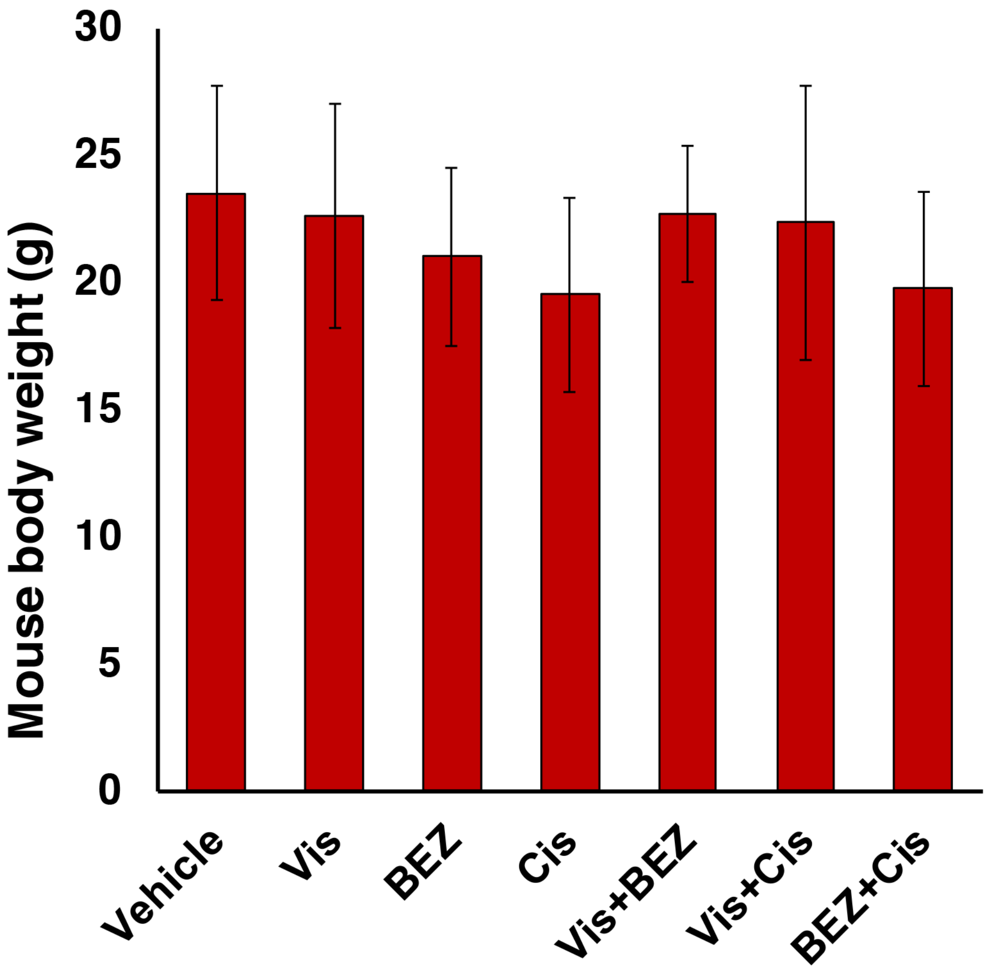

Supplementary Figure 4: Combination effects of inhibitors on body weight of the MB xenograft mice. The bar graph is Showing the mean body weight of mice on day 21 following treatment with inhibitors alone or combined as indicated.
